# Altered *Disrupted-in-Schizophrenia-1* Function Affects the Development of Cortical Parvalbumin Interneurons by an Indirect Mechanism

**DOI:** 10.1371/journal.pone.0156082

**Published:** 2016-05-31

**Authors:** Malgorzata Borkowska, J. Kirsty Millar, David J. Price

**Affiliations:** 1 University of Edinburgh Centre for Integrative Physiology, Hugh Robson Building, George Square, Edinburgh EH8 9XD, United Kingdom; 2 University of Edinburgh Centre for Genomic and Experimental Medicine, MRC Institute of Genetics and Molecular Medicine, Crewe Road, Edinburgh EH4 2XU, United Kingdom; UCL, UNITED KINGDOM

## Abstract

*Disrupted-in-Schizophrenia-1 (DISC1)* gene has been linked to schizophrenia and related major mental illness. Mouse Disc1 has been implicated in brain development, mainly in the proliferation, differentiation, lamination, neurite outgrowth and synapse formation and maintenance of cortical excitatory neurons. Here, the effects of two loss-of-function point mutations in the mouse *Disc1* sequence (Q31L and L100P) on cortical inhibitory interneurons were investigated. None of the mutations affected the overall number of interneurons. However, the 100P, but not the 31L, mutation resulted in a significant decrease in the numbers of interneurons expressing parvalbumin mRNA and protein across the sensory cortex. To investigate role of Disc1 in regulation of parvalbumin expression, mouse wild-type Disc-1 or the 100P mutant form were electroporated *in utero* into cortical excitatory neurons. Overexpression of wild-type Disc1 in these cells caused increased densities of parvalbumin-expressing interneurons in the electroporated area and in areas connected with it, whereas expression of Disc1-100P did not. We conclude that the 100P mutation prevents expression of parvalbumin by a normally sized cohort of interneurons and that altering Disc1 function in cortical excitatory neurons indirectly affects parvalbumin expression by cortical interneurons, perhaps as a result of altered functional input from the excitatory neurons.

## Introduction

Psychiatric disorders such as schizophrenia, bipolar disorder and major depression have a relatively high incidence in the population [1–2]. Both environmental and genetic factors contribute to their aetiology [3–4]. The genetic contribution is complex. Disrupted-In-Schizophrenia-1 (*DISC1*) is one gene whose loss-of-function has been associated with major psychiatric illness [5–9]. DISC1 is an 854 amino acid coiled-coiled scaffold protein that interacts with other proteins such as PDE4B, NDE1/NDEL1 and GSK3β, which have themselves been linked to psychiatric disorders [10–12].

A number of studies have used mutant mice to test the cellular functions of Disc1. Disc1 is expressed broadly throughout the developing and adult brain [13–14] and is implicated in neurogenesis, neuronal migration, neurite outgrowth, and synapse formation and maintenance [10, 15, 12, 16–20]. Mice carrying point mutations in exon 2 of *Disc1* (known as L100P and Q31L) are of particular interest since some studies have shown that they display schizophrenia-like and depression-like behaviours that can be reversed using antipsychotics and antidepressants ([17, 21–23]; see [24] for cautionary findings). These mutations interfere with Disc1 functions such as binding to Pde4b [17], and mice carrying them show disrupted radial migration of cortical projection neurons and a reduced number of cortical neurons. 100P mice have a deficit in dendritic spine length and density [25–26]. Relatively little is known about inhibitory interneuron generation in these mutants, but this is of great interest since, in humans, there is increasing evidence that dysfunction of cerebral cortical inhibitory circuits is a major cause of cognitive defects in schizophrenia [27–28]. Evidence from post-mortem studies showed a decrease in the numbers of specific types of cortical interneurons, namely parvalbumin, somatostatin and calbindin-containing cells, in patients with schizophrenia or bipolar disorder [29–34].

Given the widespread expression of Disc1 across the cortex, we tested whether 100P and 31L mutations cause widespread defects in cortical interneuron populations. We found a decrease of parvalbumin expression by cortical interneurons across several major sensory areas in 100P mutants. We then used *in utero* electroporation to test whether parvalbumin expression by cortical interneurons, which arise subcortically and migrate to the cortex [35–37], might be affected indirectly by changes in Disc1 expression in cortical pyramidal cells.

## Materials and Methods

### Mice

Mice were maintained and bred and all procedures were carried out as stipulated by Home Office UK regulations The licences authorising this work where approved by the University of Edinburgh’s Ethical Review Committee and the Home Office. Animal husbandry was in accordance with the UK Animals (Scientific Procedures) Act 1986 regulations.

ENU mutant mice (characterised in [17]) of mixed sexes were bred in-house on a c57Bl/6 background to generate 100P/+, 100P/100P, 31L/+ and 31L/31L mice with their wild-type (WT) littermates. Mice were sacrificed at postnatal day 21 (P21) and sequenced for the mutation (described in [17]). Females used for the *in utero* experiments were obtained from a cross between C57Bl/6J males and CBA females that had been bred in-house. The day of the vaginal plug was considered as E0.5. Brain tissue of the 100P and 31L heterozygous and homozygous mutants and their WT littermates came from animals of similar weight (+/- 0.5g from the litter’s mean weight).

### Expression plasmids

Mouse coding sequences for WT *Disc1* or for the mutant 100P form of *Disc1* conjugated to an N-terminal FLAG-tag were subcloned from pcDNA-4TO® (Invitrogen) into pCAGGS-IRES-(NLS) eGFP [38] for use in the *in utero* electroporation experiments. To examine long range connections established by electroporated cells, Tau-GFP plasmid pTP6 [39] was co-electroporated with the above constructs.

### *In utero* electroporation

Pregnant dams (E14.5) were anaesthetised with 3% isofluorane (Merial) mixed with oxygen. Depth of anaesthesia was controlled by checking involuntary reflexes (toe or tail pinch reflex) and monitored throughout the surgery. To prevent heat loss, the anaesthetised animal was placed on a heating pad. Prior to the procedure, all animals were given a subcutaneous injection of 0.05 mg/kg Buprenorphine (Vetergesic, Reckitt Benckiser Healthcare) dissolved in water for injections (Norbrook). The fur on the abdomen was shaved off with an electric trimmer and sterilised with a solution of MediScrub (MediChem) in water. A 1–1.5 cm incision was made through skin with sterile scissors, the abdominal muscles were cut along the midline and the uterine horns were exposed. The embryos were taken out three at a time and kept moist with a pre-warmed solution of sterile phosphate buffered saline (PBS). 2–3 μg (~2μg/μl) of DNA mixed with Fastgreen dye (Sigma) was injected into each embryo’s lateral ventricle. Forceps-type electrodes (Nepagene, CUY650P5) were placed around the target region with the positive electrode at the targeted area [40] and five 50ms pulses of 35V were passed through them. The embryos were replaced in the abdomen, which was closed with grade 5 vicryl surgical sutures (Ethicon). The skin was clipped with 7mm Reflex Clips (WPI, 500344) using Reflex Clip Applier (WPI, 500343) and the pregnant female was placed in a clean cage on a heating mat to recover. Post-operative analgesia in the form of buprenorphine jelly was administered orally at 0.5mg/kg and daily checks were made to monitor the recovery. The survival rate of females was nearly 100%, while that of their embryos varied from 70% to 100%.

### Tissue preparation

ENU mutant and electroporated mice were sacrificed by overdose of anaesthetic (0.2ml of 200mg sodium pentobarbital) at P21. ENU mutants were perfused with cold PBS and 4% paraformaldehyde (PFA), while electroporated animals were perfused with ice cold PBS only, checked for GFP expression and then fixed overnight at 4°C with 4% PFA. Tissue was then cryoprotected in 30% sucrose, embedded in 15% sucrose/Lamb's OCT (Thermo Scientific) and cut into 20μm sections.

For the analysis of interneuron density in the ENU mutant cerebral cortex, as well as parvalbumin expression in the in utero overexpression experiment, 240 consecutive 20 μm sections were taken starting from the first sighting of the narrowed and arched anterior forceps of the corpus callosum above the lateral ventricle. Sections from different genotypes were matched based on anatomical features. For the analysis of interneuron density in the medial prefrontal cortex, 20 μm sections were cut for each genotype starting at the first appearance of the lateral ventricle and anterior forceps of the corpus callosum, as well as the disappearance of the medio-lateral ‘tract’ in the molecular layer of the piriform cortex.

### Immunohistochemistry

Sections were washed in PBS and subjected to antigen retrieval by washing and boiling in 10mM sodium citrate buffer (pH6) for 10 min and 20 min respectively (5 min boil for GAD67 staining). The sections were incubated in 0.1% Triton X 100, 0.2% gelatin from cold water fish skin (Sigma-Aldrich) and 10% goat, donkey or equine serum, or in 5–10% skimmed milk (for GAD67 staining) for 2h at room temperature. Primary antibodies were diluted in 0.1% Triton X 100, 0.2% gelatin and 5% appropriate serum: mouse monoclonal anti-parvalbumin (1:1000, P3088 Sigma-Aldrich), rat polyclonal anti-somatostatin (1:200, AB5494 Millipore), rabbit monoclonal anti-calretinin (1:1000, CR7697 Swant), rabbit polyclonal anti-GAD67 (1:100, MAB5406 Millipore), polyclonal goat anti-GFP (1:500, ab6673 Abcam), rabbit polyclonal anti-Flag (1:500–1:50, F7425 Sigma-Aldrich). The sections were incubated with primary antibodies at 4°C overnight then with secondary antibody at room temperature for 1h. Sections were stained for nuclear marker DAPI (1:50000, Invitrogen) and mounted with Vectashield HardSet (Vector Laboratories). If 3,3'-diaminobenzidine (DAB) staining was performed, after incubation with the primary antibody endogenous peroxidases were blocked with 3% H_2_O_2_/10% methanol in PBS for no longer than 30 mins at room temperature. After secondary antibody incubation sections were incubated in the avidin-biotin solution (ABC, Vector Laboratories) for 1h at room temperature. After washing, staining with 0.05% DAB in Tris buffer saline (Vector Laboratories) was performed at room temperature and slides were mounted with Aquatex mounting medium (Merck).

### *In situ* hybridisation

A parvalbumin RNA probe was generated as described in [41] labelled with digoxigenin (DIG) (Roche) and used at 1:1000 dilution. The probe was denatured for 10 min at 85°C and sections were incubated with it overnight at 65°C in a pre-warmed humidified container. Sections were washed in maleic acid buffer, blocked in maleic acid buffer with sheep serum and 2% blocking reagent (Roche) for 1h at room temperature and then incubated overnight with an alkaline phosphatase (AP)-tagged anti-DIG antibody (1:500, Roche) at 4°C. Colorimetric reaction was performed with NBT/BCIP (Roche) and the sections were mounted with Aquatex mounting medium (Merck).

### Analysis

**Fig 1:**
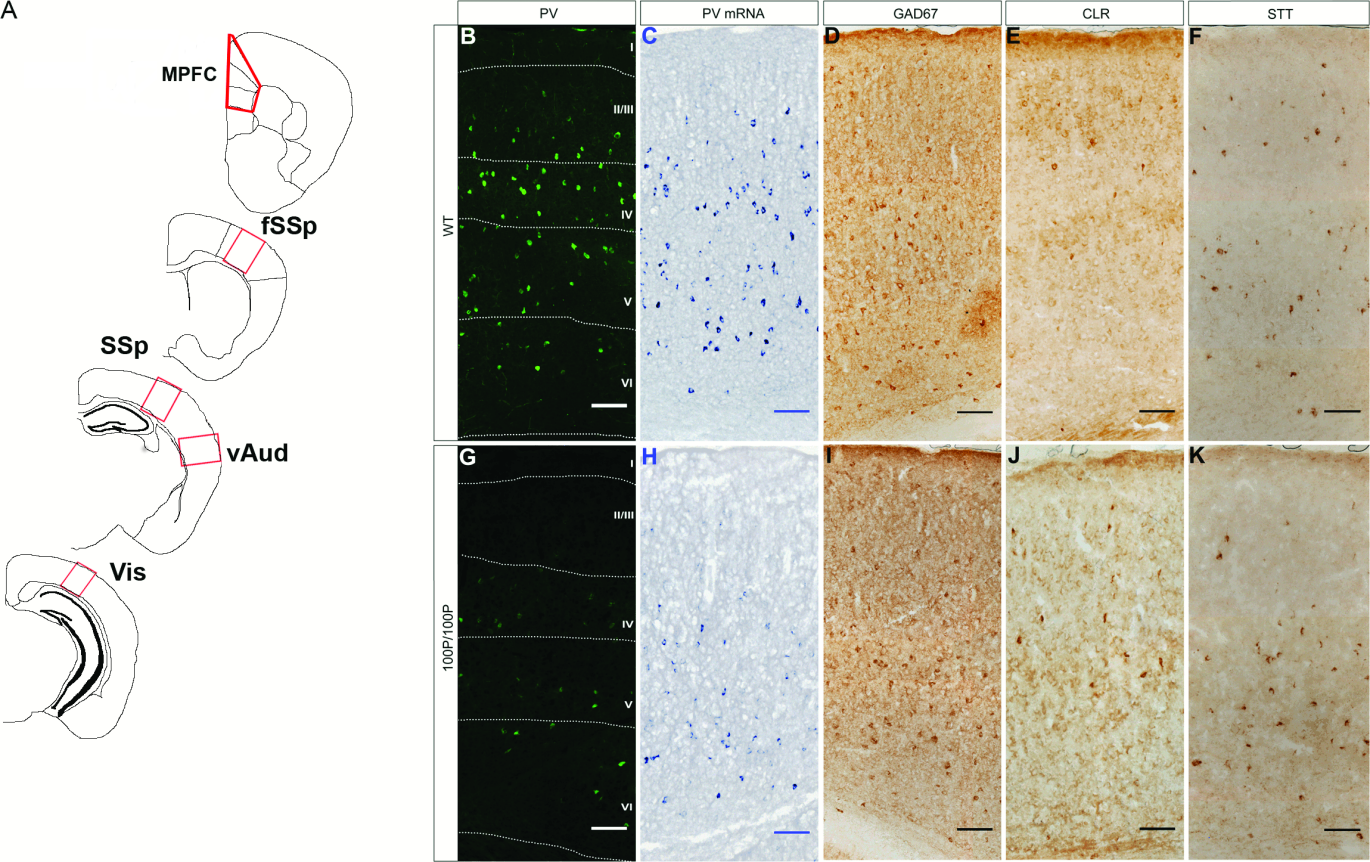
Analysis of interneurons in the cortex of wild-type (WT) and mutant mice. (**A**) Coronal sections at P21 with regions of interest marked by red boxes: medial prefrontal cortex (MPFC), frontal primary somatosensory cortex (fSSp), primary somatosensory cortex (SSp), ventral auditory cortex (vAud) and primary visual cortex (Vis). (**B-K**) Representative images of interneuronal marker expression in SSp in WT and 100P/100P mice. Abbreviations: PV, parvalbumin; GAD67, glutamate decarboxylase 67; STT, somatostatin; CLR, calretinin. Cortical layers marked in B and G are in approximately the same positions in the other images. Scale bars: 100μm.

Sectioning, staining, imaging and counting were done blind to genotype. Images were reconstructed in Adobe Photoshop (CS2 version 9) and analysed using ImageJ (Rasband, W.S., ImageJ, U. S. National Institutes of Health, Bethesda, Maryland, USA, http://imagej.nih.gov/ij/, 1997–2011). For GAD67 staining, analysis was performed using a camera lucida. Regions of interest (ROI) were selected using anatomical and cytoarchitectural features to define major cortical areas (Fig 1A). In short, primary somatosensory cortex (barrel cortex) was distinguished based on the characteristic appearance of layer 4 (i.e. its readily discernible well-described barrel structure). For frontal somatosensory cortex, the counting grid was moved directly anterior to the barrel cortex. For the visual cortex, the counting grid was placed in posterior cortex above the dorsal end of the dentate gyrus. For the ventral auditory cortex, the counting grid was placed dorsal to a characteristic indentation in the most lateral part of the cortex beneath which there is a change in the layer cytoarchitecture at the border between auditory and entorhinal cortex. For the medial prefrontal cortex, the ventral border of the infralimbic cortex was placed at the dorsal edge of the dorsal peduncular area, about one third of the distance up the medial edge of the anterior forceps of the corpus callosum. The prelimbic cortex was distinguished from the infralimbic cortex since it has a higher cell density. A line running along the border of the anterior cingulate area and secondary motor cortex was placed at ~50° to the horizontal starting from the dorso-medial corner of the anterior forceps of the corpus callosum. Cortical landmarks were equally visible in all mice irrespective of genotype or treatment. Systematic random sampling [42–43] was used to section through each ROI and 500 μm wide counting boxes were aligned with the pia and stretched to include all cortical layers. Their bases were placed along the border of the white and grey matter. Data from interneuron marker analysis of the ENU mutants were normalised to the total number of cells marked by a nuclear stain (DAPI) and presented as a proportion [%]. Cells with visible nuclei were counted. Profile counts were used uncorrected since there were no detectable differences in cell sizes or shapes between genotypes or treatment groups (i.e. the principle of Equal Opportunity of Detection was applied: [43]).

Analysis of the interneurons density in the neocortical ROI as a whole was performed using an ordinary one-way ANOVA to assess the effects of ENU mutations. For subsequent analyses, data from each ROI were kept separate and ordinary two-way ANOVA was used to test the effects of genotype/ electroporated construct and location. ANOVAs were followed by planned within group comparison where applicable. Post-hoc tests were used where ANOVA showed significant variation with the alpha of 0.05 and the probabilities were routinely corrected for multiple testing. For analysis of ENU mutants, the experiments were designed to compare heterozygous and homozygous 100P mutants with their WT littermates and heterozygous and homozygous 31L mutants with their WT littermates. Analyses were done using GraphPad Prism® 6.0 (GraphPad Software, San Diego, California, USA).

## Results

### *Disc1* 100P but not 31L mutation decreases parvalbumin expression in cortex

**Fig 2:**
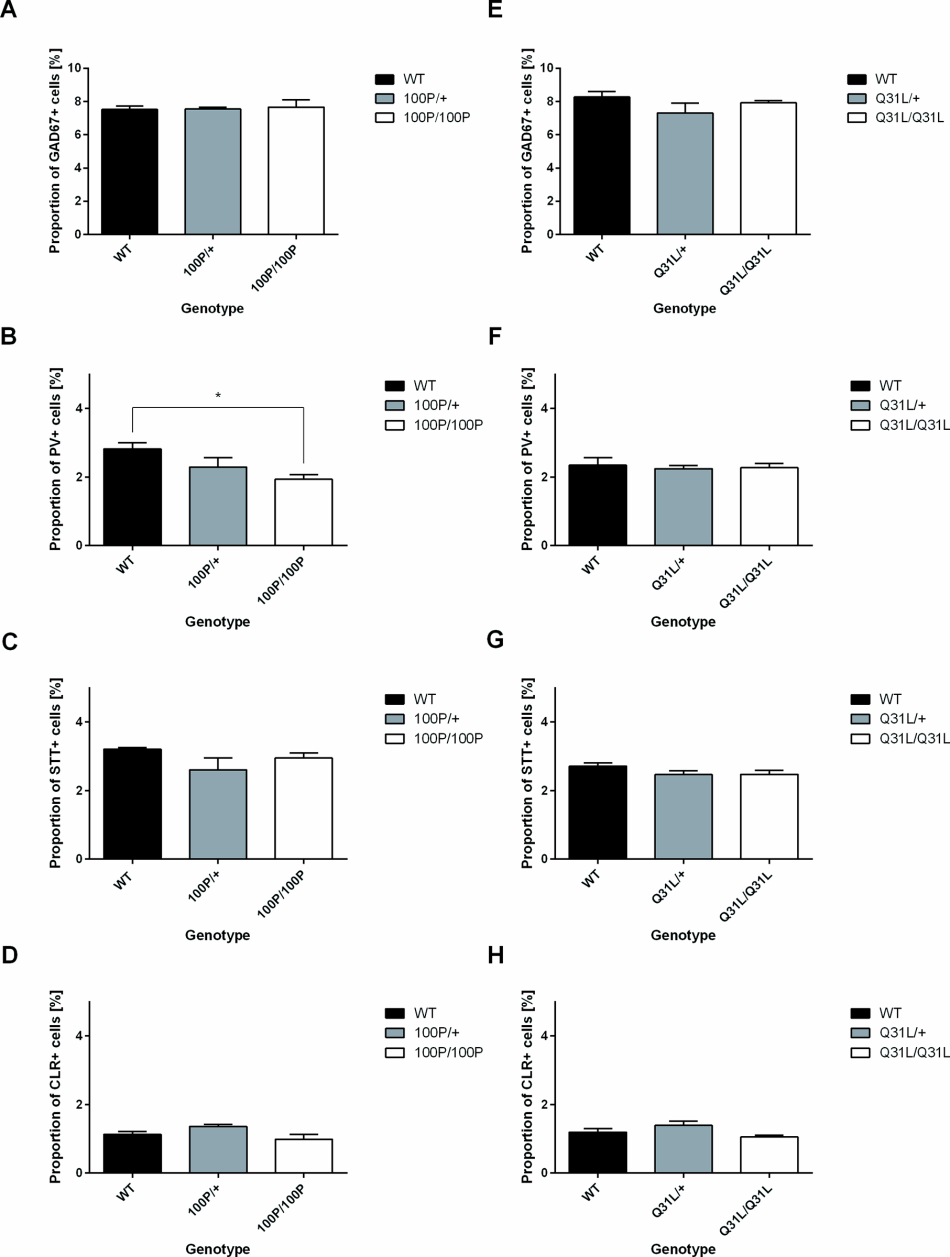
100P homozygous mice show overall decrease in proportions of PV-expressing cells in the P21 neocortex. (**A, B**) Proportion of PV-expressing cells was significantly decreased in the 100P/100P mouse cortex without any loss in the total interneurons number. (**C, D**) No difference was observed in other two subpopulations of cortical interneurons: STT- and CLR-positive cells (n = 3–8 animals for each comparison; data are means ± sems; * p<0.05, ** p<0.01, one-way ANOVA with Bonferroni’s multiple comparisons test where applicable). (E-H) No change in the PV, GAD67, CLR and SST was observed in the 31L mutant mice neocortex (n = 3–9 animals, one-way ANOVA). Full ANOVA results are in S1 Table.

We assessed if a point mutation in the *Disc1* gene results in changes in proportions of interneurons across the neocortex as a whole, using the general interneuron marker glutamate decarboxylase 67 (GAD67) and markers of specific subpopulations, parvalbumin (PV), calretinin (CLR) and somatostatin (SST) [44–45] (Fig 1B–1K). One-way ANOVA showed no significant effects of 100P genotype on the overall proportions of interneurons as assessed by GAD67 staining (Fig 2A, n = 5 WT, n = 4 100P/+, n = 3 100P/100P; results of ANOVA can be found in S1 Table). An effect of 100P genotype on the proportions of PV expressing cells was observed in 100P/100P mice as compared to their WT littermates (Fig 2B, n = 8 WT, n = 4 100P/+, n = 4 100P/100P; one-way ANOVA, F(2, 13) = 5.282, p = 0.0209). No significant 100P genotype effects were observed on either of the other two major cortical interneurons subpopulations (Fig 2C and 2D; S1 Table; SST: n = 5 WT, n = 3 100P/+, n = 3 100P/100P; CLR: n = 5 WT, n = 3 100P/+, n = 3 100P/100P). There were no significant effects of 31L genotype on any of the interneuron markers used (Fig 2E–2H, S1 Table; GAD67: n = 4 WT, n = 3 31L/+, n = 4 31L/31L; PV: n = 7 WT, n = 3 31L/+, n = 9 31L/31L; STT: n = 7 WT, n = 3 31/+, n– 7 31L/31L; CLR: n = 4 WT, n = 3 31L/+, n = 4 31L/31L). These results indicate a specific effect of 100P mutations on expression of PV by the interneuron population rather than an overall reduction in the proportion of interneurons.

**Fig 3:**
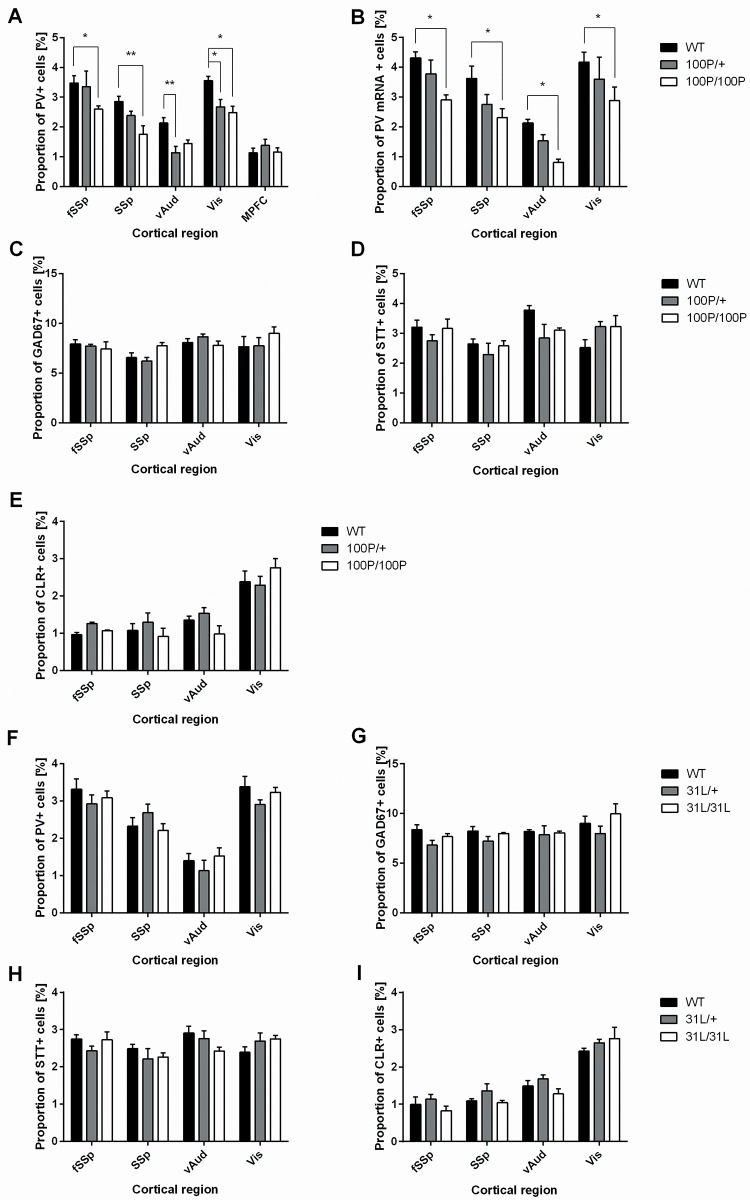
Mice carrying 100P mutations show region-specific decreases in proportions of PV-expressing cells. (**A**) Significant decreases in the proportions of PV+ cells were observed in fSSp, SSp, vAud and in Vis, but not MPFC. (**B**) Corresponding decreases in the proportions of cells expressing the mRNA for PV were observed in fSSp, SSp and vAud. (**C**) There were no corresponding changes in the proportions of GAD67+ cells. (**D**, **E**) No major change was observed in the proportion of cells expressing calretinin (CLR) or somatostatin (STT) across the mutant cortex (n = 3–8 animals for each comparison; data are means ± sems; * p<0.05, ** p<0.01, two-way ANOVA with Dunnet’s multiple comparisons test where applicable). (**F**-**I**) No change in the PV, GAD67, CLR and SST was observed across the cortices of the 31L mutant mice (n = 3–9 animals, two-way ANOVA with Dunnet’s multiple comparisons test where applicable). S2 Table provides the results of ANOVA.

Our experiments were designed to investigate the possibility that Disc1 mutation might have effects that depend on neocortical region. Exploratory analyses were carried out in (i) the medial prefrontal cortex (MPFC), (ii) the frontal primary somatosensory (fSSp) cortex, (iii) the primary somatosensory (SSp; barrel) cortex, (iv) the ventral auditory (vAud) cortex and (v) the visual (Vis) cortex in the two *Disc1* mutant mouse strains at P21 (Fig 1A). The proportions of cortical cells expressing GAD67, PV, SST and CLR were counted in sampling areas spanning the full cortical depth (Fig 1). Genotype × ROI ANOVA with data from the 5 cortical regions showed a significant main effect of genotype but no genotype × ROI interaction (Fig 3A; two-way ANOVA, F(2, 55) = 11.82, p<0.0001). Post-hoc tests found evidence that mean proportions of cells expressing PV protein were lower in 100P/+ and/or 100P/100P mutants than in wild-types (WTs) in fSSP, SSP, vAud and Vis areas (Fig 1B and 1G; Fig 3A). Differences between WTs and 100P/100P mice were significant in fSSp and SSp cortices (Fig 3A; n = 8 WT, n = 4 100P/+, n = 4 100P/100P; Dunnet’s multiple comparisons test, fSSp: p = 0.0133; SSp: p = 0.0017). A significant difference in PV cells was also observed in the Vis cortex of 100P/+ and 100P/100P when compared to their WT littermates (Fig 3A; n = 3 WT, n = 4 100P/+, n = 3 100P/100P; Dunnet’s multiple comparisons test, WT *vs* 100P/+ p = 0.0229; WT *vs* 100P/100P: p = 0.0497). In the vAud cortex, a significant difference in PV expression was observed between WTs and 100P/+ mice (Fig 3A; n = 8 WT, n = 4 100P/+, n = 4 100P/100P; Dunnet’s multiple comparisons test, p = 0.0045). A decrease in PV cells density in the 100P/100P vAud cortex was observed, but it did not reach significance (Dunnet’s multiple comparisons test, p = 0.0582). Overall, these results indicate that the 100P mutation causes reductions in the proportions of PV cells of varying magnitude across sensory cortex. There was no significant genotype effect on proportions of PV cells in MPFC (Fig 3A; Dunnet’s multiple comparisons test, WT *vs* 100P/+: p = 0.9982; WT *vs* 100P/100P: p = 0.7557). Therefore, this region was not included in further analysis. ANOVA also showed effects of region on proportions of PV cells; the full results of ANOVA are shown in S2 Table.

To assess whether the observed reductions in proportions of PV-expressing cells were due to a transcriptional defect, we performed *in situ* hybridisations with a PV probe [41]. ANOVA showed significant effects of genotype on proportions of PV mRNA+ cells in fSSp, SSp, vAud and Vis cortices (two-way ANOVA, F(2, 36) = 13.40, p<0.0001). In fSSp, SSp, vAud and Vis cortices, the mean proportions of cells expressing PV mRNA were lower in 100P/+ and 100P/100P mutants than in wild-types (WTs), reflecting the results obtained by testing for PV protein (Fig 1C and 1H; Fig 3B). Significant reductions in 100P/100P mice in fSSp, SSp, vAud and Vis cortices (Dunnet’s multiple comparisons test, n = 4 for each region and genotype; fSSp: p = 0.019; SSp: p = 0.028; vAud: p = 0.02744; Vis: p = 0.031) were of similar magnitude to those observed when cells expressing PV protein were counted (Fig 3A). We conclude that there is a major effect of 100P mutation on the proportions of cells expressing PV mRNA in fSSp, SSp,vAud and Vis cortices. Region effects are reported in S2 Table; there were no interaction effects.

To investigate if the decrease in the proportion of PV-expressing cells in mice carrying 100P mutations reflected an overall decrease in the proportions of cortical interneurons in any of the affected regions, we analysed the proportions of GAD67-positive cells in fSSp, SSp, vAud and Vis cortices. There were no significant effects of genotype (Figs 1D and 1I; 3C; WT: n = 5 (4 for Vis); 100P/+: n = 4; 100P/100P: n = 3; results of ANOVA are in S2 Table), indicating that 100P mutations cause a loss of expression of PV by the interneuron population rather than a loss of interneurons.

The proportions of cells that fell into two other major subpopulations of interneurons, namely SST+ and CLR+, were unaffected by 100P mutations (Fig 1E, 1F, 1J and 1K; Fig 3D and 3E; WT: n = 5 (3 for Vis); 100P/+ and 100P/100P: n = 3, results of ANOVA are in S2 Table).

In our analysis of 31L mice, ANOVA showed no significant effects of genotype on the proportions of interneurons expressing PV, GAD67, SST or CLR (Fig 3F; WT: n = 7 (4 for Vis); 31L/+: n = 3; 31L/31L: n = 9 (5 for Vis)) (Fig 3G; WT: n = 4; 31L/+: n = 3; 31L/31L: n = 4 (5 for Vis)) (Fig 3H; WT: n = 4 (5 for Vis); 31L/+: n = 3; 31L/31L: n = 4 (5 for Vis)) (Fig 3I; WT: n = 7 (6 for Vis); 31L/+: n = 3; 31L/31L: n = 8 (5 for Vis); results of ANOVA are in S2 Table). Similar region effects to those observed in 100P mutants were observed and there were no interaction effects (S2 Table contains full details of ANOVA results).

These findings indicate that the 100P *Disc1* mutation affects the transcription of PV by cortical interneurons that populate several major sensory areas of the cortex. A different *Disc1* mutation, 31L, does not have these effects.

### Layer-dependent effects on cortical interneurons in 100P/100P mice

**Fig 4:**
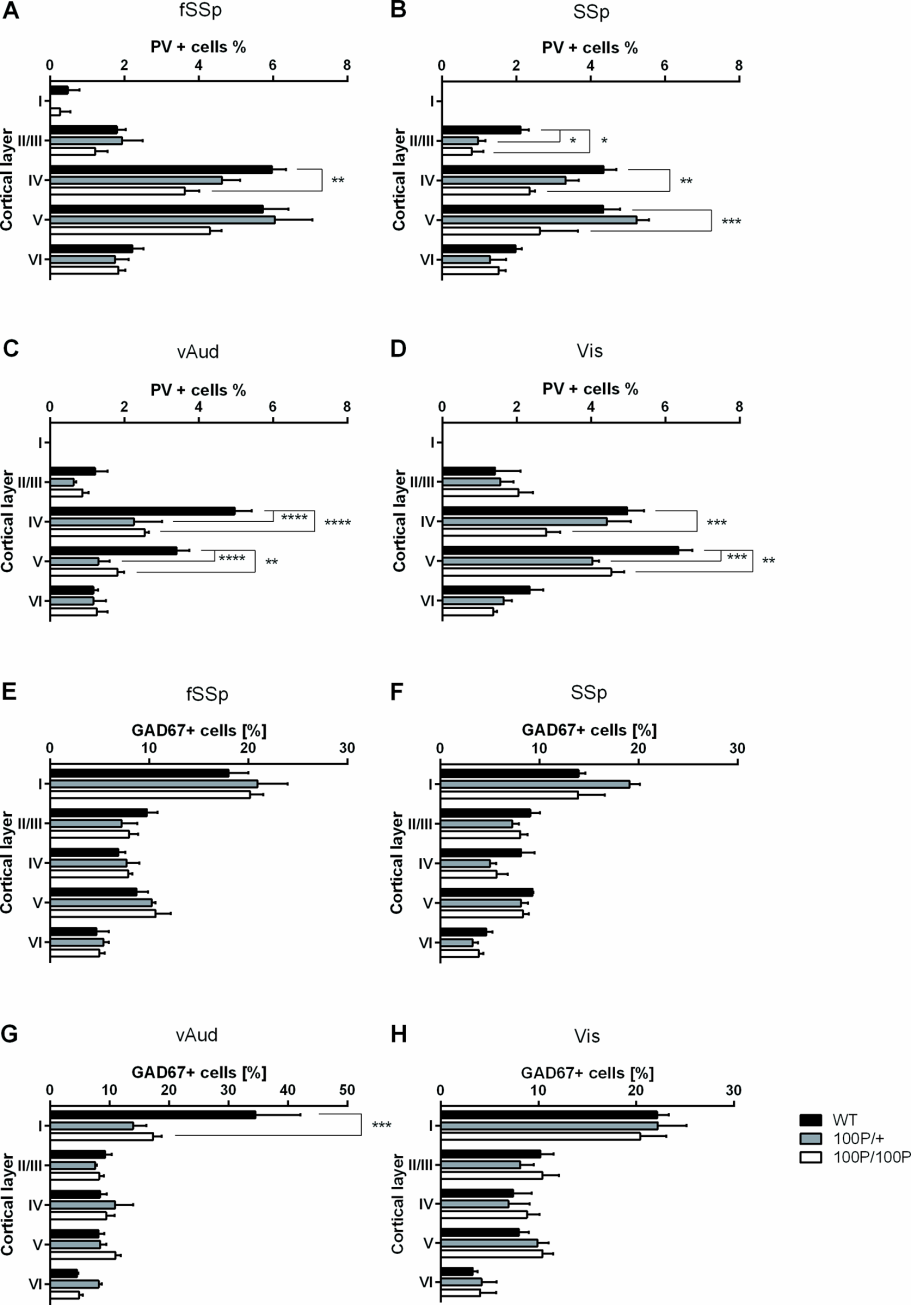
Mice carrying 100P mutations show layer-specific decreases in proportions of PV+ cells. (**A-D**) Decreases occurred in layer IV in fSSp, SSp, vAud and Vis, in layer V in SSp, vAud and Vis and in layer II/III in SSp. (**E-H**) For GAD67+ cells, the only differences were in layer I in vAud. (n = 3–8 animals; data are means ± sems; * p<0.05, ** p<0.01, *** p<0.001, two-way ANOVA with Dunnet’s multiple comparisons test where applicable). S3 Table provides the results of ANOVA.

Having shown an effect of 100P mutation on PV expression, we explored our data further to discover whether this effect is stronger in some cortical layers than others. The proportions of PV cells were counted in each layer in fSSp, SSp, vAud and Vis cortices (Fig 4). ANOVA showed a significant effect of cortical layer on the proportions of PV cells (Fig 4A–4D; S3 Table); PV+ cells were concentrated in layers IV and V. Effects of genotype were significant in all four regions and there were significant interaction effects between genotype and cortical layer in SSp, vAud and Vis (S3 Table). In all four cortical regions there was a substantial reduction in the proportion of PV-expressing cells in cortical layer IV in 100P/100P mice compared to WT littermates (Fig 4A–4D; numbers of animals as for data in Fig 3; Dunnet’s multiple comparisons test, fSSp: p = 0.0012; SSp: p = 0.0003; vAud: p < 0.0001; Vis: p = 0.0006). A significant decrease in PV expression in layer IV of vAud cortex was also observed in 100P/+ mice (Fig 4C; Dunnet’s multiple comparisons test, p < 0.0001). In SSp, vAud and Vis cortices there was also a significant decrease in the proportions of PV cells in layer V of 100P/100P mice (Fig 4B–4D; Dunnet’s multiple comparisons test, SSp: p = 0.0019; vAud: p = 0.0.002; Vis: p = 0.0042) and, in vAud and Vis cortices, of 100P/+ mice (Fig 4C and 4D, Dunnet’s multiple comparisons test, vAUd: p <0.0001; Vis: p = 0.0001). The proportions of PV cells in layer II/III were significantly affected only in the SSp cortex of 100P/+ and 100P/100P mice (Fig 4B; Dunnet’s multiple comparisons test, 100P/+: p = 0.043; 100P/100P: p = 0.018) while proportions of PV cells in layer VI were not significantly affected in any of the four cortical regions (Fig 4A–4D). This analysis suggests that 100P mutations affect PV cell proportions primarily in layers IV and V.

Since Disc1 has a known role in the interneuronal migration [48] we investigated whether 100P mutations cause any disturbance in the laminar distribution of interneurons by analysing the proportions of GAD67+ cells in each layer in fSSp, SSp, vAud and Vis cortices (Fig 4E–4H). The only region in which ANOVA showed a marginally significant effect of genotype was vAud (F(2, 45) = 3.343, p = 0.0443; S3 Table). Post-hoc testing showed a significant difference in layer I of 100P/100P vAud cortex (Fig 4G; Bonferroni multiple comparisons test, p < 0.0001). This single marginally significant effect would need to be examined further before any robust conclusions can be drawn.

In summary, we have little or no compelling evidence that 100P mutation affects the overall proportions of interneurons populating sensory cortices and their distributions across the cortical layers. Our findings do provide robust evidence that the proportions of these neurons that express PV are significantly reduced, mainly in layers IV and V where their proportions are normally greatest.

### No detectable effects of Disc1 or Disc1-100P overexpression on pyramidal cells in P21 cortex

**Fig 5:**
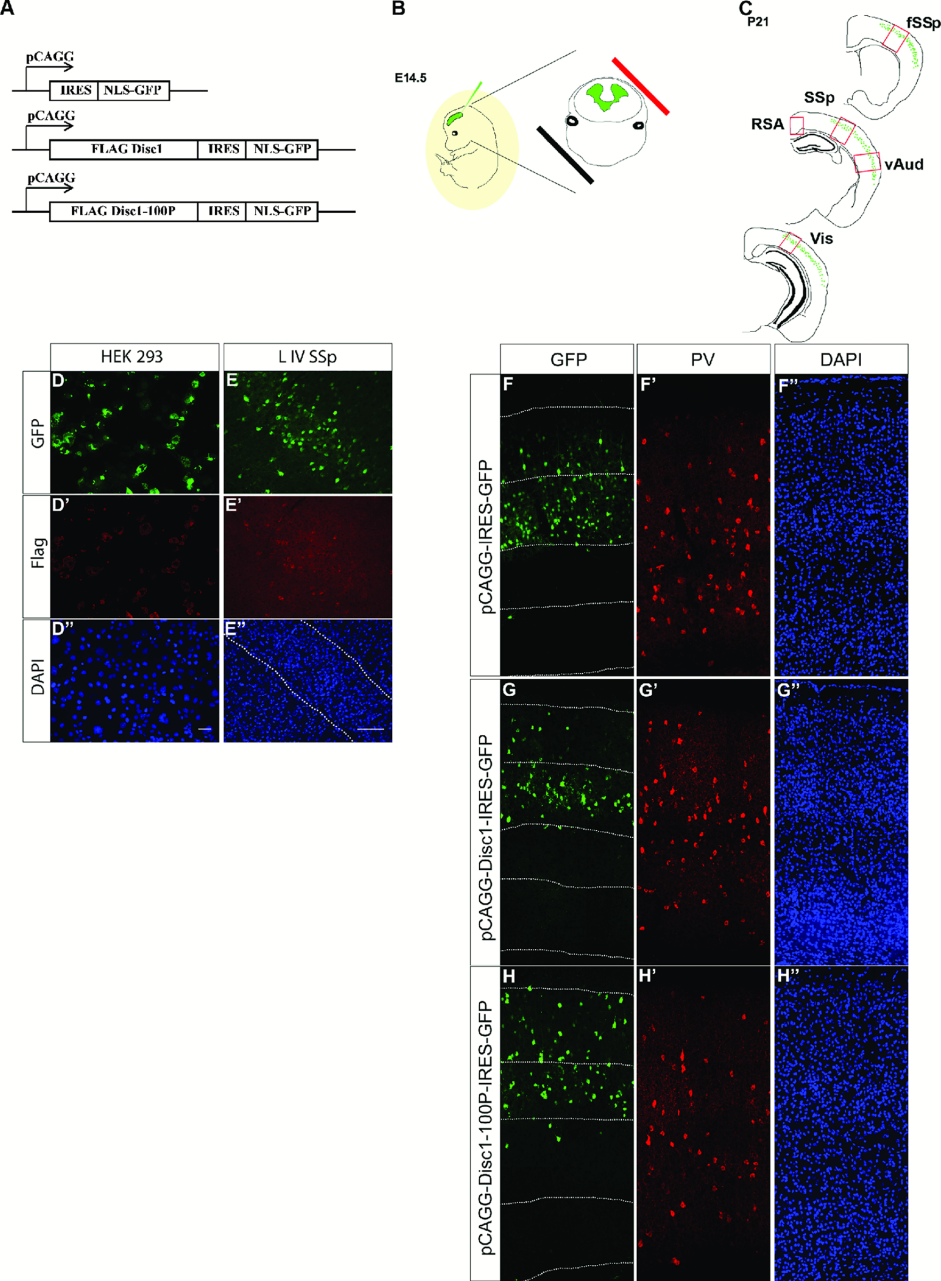
*In utero* electroporation of Disc1 and Disc1-100P constructs into wild-type neocortex and analysis at P21. (**A**,**B**) Flag-tagged Disc1 and Disc1-100P coding sequences subcloned into an IRES-NLS-GFP vector as well as the empty IRES-NLS-GFP vector were electroporated at E14.5 into cortical regions whose proportions of PV+ cells are affected in mice carrying 100P mutations. (**C**) PV cells density was analysed at P21 in in the cortical regions marked. Abbreviations in Fig 1; RSA, retrosplenial area. (**D-E”**) Expression of the constructs was assessed (**D-D''**) 2 days after transfection *in vitro* and (**E-E''**) at P21 *in vivo* (**F-H''**) GFP expression was observed predominantly in layers II/III and IV at P21 as seen in the representative images of the electroporated SSp. Scale bar: for **D-D''** 50 μm; for **E-H''** 100μm.

Our evidence indicates that the 100P mutation in *Disc1* does not prevent the cortex acquiring a normal complement of interneurons, but it reduces the proportion of interneurons expressing PV. The onset of PV expression in the rodent cortex occurs during the second postnatal week and is heavily dependent on neural activity [46–47]. This raised the possibility that altered Disc1 function might affect the proportions of PV-expressing cortical interneurons indirectly as a result of primary changes in interacting excitatory cells. Since cortical excitatory pyramidal neurons are generated from the cortical ventricular zone whereas interneurons originate subcortically in mouse, we used *in utero* electroporation into the E14.5 ventricular zone of fSSp, SSp, vAud and Vis cortices to overexpress either FLAG-tagged WT mouse Disc1 or its Disc1-100P variant specifically in cortical pyramidal neurons in these regions (Fig 5A and 5B). FLAG-Disc1 was expressed from a bicistronic plasmid vector that also carries eGFP. Analyses were carried out in the fSSp, SSp, vAud and Vis cortices and the retrosplenial area (RSA) at P21 (Fig 5C).

Immunochemistry for FLAG and GFP showed that constructs encoding either WT Disc1, the Disc1-100P variant, or GFP alone, expressed these protein species in transfected HEK293 cells *in vitro* (Fig 5D–5D”) and in P21 postmitotic cortical neurons *in vivo* (Fig 5E–5E”, 5F–5H). *In vivo*, cells that had incorporated either an empty pCAGG-IRES-GFP plasmid or either of the Disc1-producing constructs at E14.5 resided predominantly in cortical layers II/III and IV at P21 (Fig 5F–5H), in accordance with the birthdates of most of the cells populating these layers. The numbers of cells that incorporated each of the three constructs were within similar ranges.

**Fig 6:**
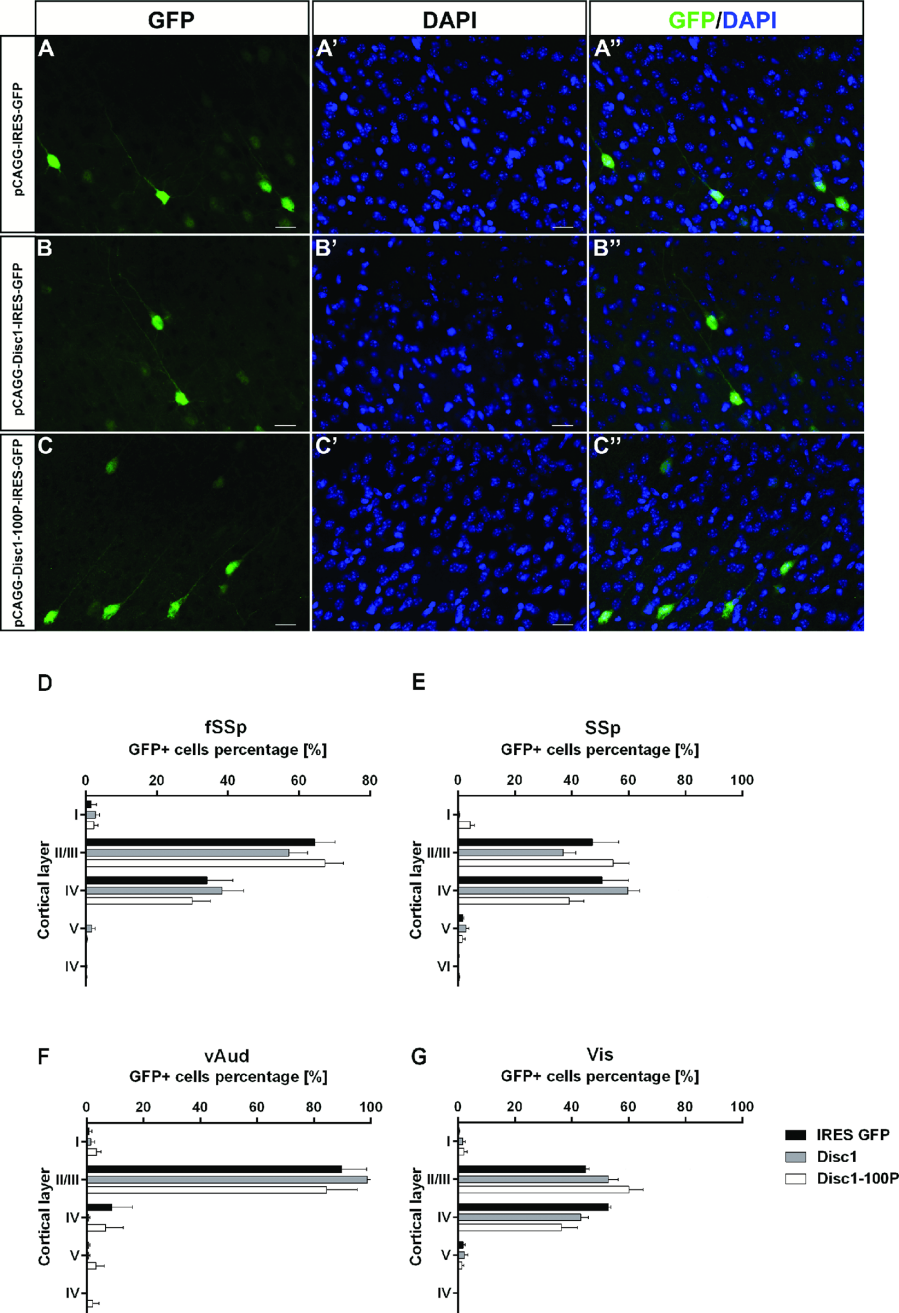
No discernible defects in the distributions of Disc1-100P overexpressing cells. (**A-C''**) Cells expressing constructs containing either no Disc1, WT Disc1 or Disc1-100P; scale bars, 50μm. (**D-G**) (n = 3–10 animals per genotype; two-way ANOVA with Bonferroni correction: * p<0.05; ** p<0.01; *** p<0.001). S4 Table provides the results of ANOVA.

Examples of electroporated cortical neurons at P21 are shown in Fig 6A–6C”; they had appearances typical of pyramidal neurons. The laminar distributions of the electroporated cells did not vary significantly with the construct used in any of the targeted cortical areas (Fig 6D–6G; two-way ANOVA results are in S4 Table; fSSp: n = 4 empty vector, n = 7 Disc1-IRES-GFP, n = 10 Disc1-100P-IRES-GFP; SSp; n = 5 empty vector, n = 7 Disc1-IRES-GFP, n = 9 Disc1-100P-IRES-GFP; vAud: n = 3 empty vector and Disc1-IRES-GFP, n = 6 Disc1-100P-IRES-GFP; Vis n = 3 empty vector and Disc1-IRES-GFP, n = 6 Disc1-100P-IRES-GFP). Overall, we found no evidence that the numbers, appearances and distributions of the electroporated cells at P21 varied between constructs.

### Effects of Disc1 or Disc1-100P overexpression on cortical PV-expressing cells

**Fig 7:**
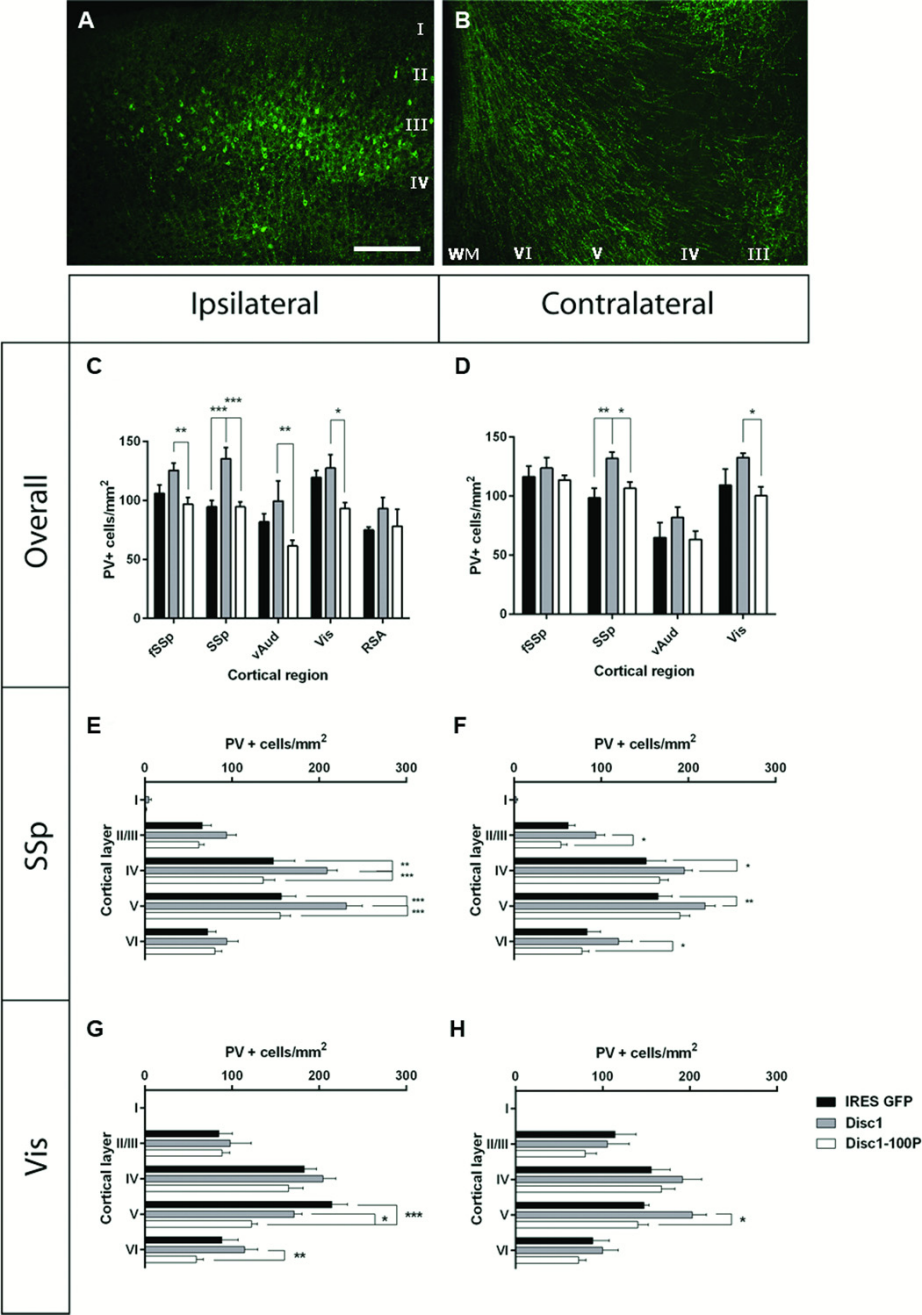
Effects of expression of Disc1 or Disc1-100P from constructs electroporated into the cortex on the densities and distributions of PV expressing cells in the P21 mouse cortex. (**A**) Cells in layer II/III targeted by the electroporation (**B**) send their callosal projections to the contralateral side. Scale bar, 150μm. (**C,E,G**) Effects on densities of PV expressing cells in (C) the electroporated cortical areas and non-electroporated retrosplenial area (RSA) and in (D,E) cortical layers in electroporated SSp and Vis. (**D,F.H**) Effects on densities of PV expressing cells in (**D**) the areas contralateral to the electroporated areas and (**F,H**) cortical layers in areas contralateral to electroporated SSp and Vis (n = 3–10 animals per genotype; multiple comparisons two-way ANOVA with Bonferroni correction: * p<0.05; ** p<0.01; *** p<0.001). S5 Table provides the results of ANOVA.

In each animal, we counted the densities of PV-expressing cells in the cortical regions that contained electroporated cells (Fig 5C and 5F’–5H”). We also counted the densities of PV-expressing cells in the contralateral non-electroporated region exactly opposite to the electroporated region, which was innervated by the callosal axons of electroporated cells (Fig 7A and 7B). As a control, we counted PV-expressing cells in the RSA ipsilateral to the electroporated hemisphere, which was never electroporated and in which we could not detect axons from electroporated regions.

In the electroporated areas, fSSp, SSp, vAud and Vis, there was a significant effect of construct type on densities of PV+ cells (Fig 7C two-way ANOVA, F(2, 60) = 20.33, p<0.0001; S5 Table). There was also an effect of region, in agreement with data presented above, but no interaction effect (S5 Table). Densities were never significantly different between animals treated with Disc1-100P and empty vector, but were always significantly higher when the WT Disc1 construct rather than the Disc1-100P construct was used (first four sets of data in Fig 7C; number of animals as stated above; Bonferroni’s multiple comparisons test, fSSp: p = 0.0029; SSp: p <0.0001; vAud: p = 0.0064; Vis: p = 0.016). In RSA ipsilateral to the electroporation, there were no significant differences between any of the treatment groups (Fig 7C). These results indicate that the overexpression of WT Disc1 by cortical pyramidal cells can increase the densities of surrounding PV-expressing cortical cells, even though these neurons do not themselves contain the electroporated constructs, and that the 100P mutation of the overexpressed Disc1 blocks this indirect effect.

Remarkably, similar effects of construct type on the PV populations were seen in contralateral cortical areas mirroring those containing electroporated cells (Fig 7D; number of animals as stated above; two-way ANOVA, F(2,54) = 9.066, p = 0.0004; S5 Table). In two areas, post-hoc test showed differences that were significant. Overexpression of WT Disc1 in SSp caused PV cell densities in the contralateral SSp to increase significantly over those recorded when empty vector or Disc1-100P was used (Fig 7D; Bonferroni’s multiple comparisons test, Disc1 *vs* Disc1-100P: p = 0.017; Disc1 *vs* empty vector: p = 0.0054). In Vis cortex, the effects of the contralateral electroporation differed between the WT Disc1 and Disc1-100P constructs (Fig 7D; Bonferroni’s multiple comparisons test, p = 0.033). These findings indicate that the indirect effects observed among the cells in the electroporated area can extend to those at a distance from, but connected to, the electroporated area.

Since electroporation affected connected populations, and connections differ between cortical layers, we went on to explore the effects of electroporation on densities of PV cells in different layers in electroporated SSp and Vis cortices and their contralateral counterparts (Fig 7E–7H). Two-way ANOVA showed effects of construct type and layer and interaction effects between these two variables in the electroporated region and showed effects of construct type and layer, but no interaction effects, in contralateral areas (S5 Table). PV+ cells were densest in layers IV and V, as observed in previous experiments. In ipsilateral SSp, PV+ density was greatest when the WT Disc1 construct was used (Fig 7E; Bonferroni’s multiple comparisons test: WT Disc1 *vs* the Disc1-100P construct, layer IV: p < 0.0001; layer V: p < 0.0001; WT Disc1 *vs* empty vector, layer IV: p = 0.0027; Layer V: p = 0.0002). In contralateral SSp cortex, the effects of the WT Disc1 construct were significantly higher than those of the Disc1-100P construct or the empty vector in layers II/III to VI (Fig 7F; Bonferroni’s multiple comparisons test: layer II/III: p = 0.017; layer IV: p = 0.025; layer V: p = 0.0042; layer VI: p = 0.011). In ipsilateral Vis cortex, the effects of the WT Disc1 *vs* the Disc1-100P construct were significantly different in layers V and VI (Fig 7G; Bonferroni’s multiple corrections test layer V: p = 0.021; layer VI: p = 0.0077) although, unusually, the highest density in layer V was found when the empty vector was used (this density was not significantly different to that found when WT Disc1 was used and its significance is unclear). In contralateral Vis cortex, the effects of the WT Disc1 *vs* the Disc1-100P construct were significantly different in layer V (Fig 7H; Bonferroni’s multiple comparisons tests, p = 0.011). The results of this laminar analysis suggest that the effects of electroporation with WT Disc1 are fairly widespread across the ipsilateral and contralateral layers but greater statistical power is required to confirm the details.

In summary, the statistically robust outcomes of this analysis indicate that WT Disc1 overexpression by cortical pyramidal cells, but not the 100P form of Disc1, can increase the densities of PV-expressing cortical interneurons that are likely to be connected to the overexpressing cells. The overall picture is that *Disc1* function can affect PV expression in an indirect, cell non-autonomous manner.

## Discussion

The aim of this study was to investigate the role of Disc1 in cortical interneuron development. We analysed the density and distribution of different subpopulations of cortical interneurons in two mouse strains with ENU-induced point mutations in *Disc1*: L100P and Q31L. The main interneuron defect identified was a widespread decrease in the size of the PV interneuron population across cortical sensory regions in mice carrying 100P mutations, but not in those carrying 31L mutations. This difference in the comparison of the mutant strains and their WT littermates is interesting, adding further to the evidence described by Clapcote et al. (2007) [17] that the strains show different behavioural abnormalities and responses to antipsychotic and antidepressant drugs. In the light of our new findings, one possible reason for differences between the mutant strains in behavioral defects and/or drug responses is that only the 100P mutation affects PV expression. This might be due to variation in the way in which the mutations affect Disc1’s ability, as a scaffold protein, to interact with its binding partners such as Pde4b, a cyclic AMP-specific phosphodiesterase, whose binding was shown to be markedly reduced by the 100P mutation and to some extend by the 31L, *in vitro* [17]. Such variations might cause each mutation to affect neuronal function and hence PV expression differently.

In mice carrying the 100P mutation, we found a decrease in the expression of both PV mRNA and protein by cortical interneurons with no effect on the overall size of the interneuron population. In mice, interneurons are generated in subcortical regions including the ganglionic eminences, from where they migrate to the cerebral cortex [35–37]. Previous work using other methods to affect Disc1 activity have shown that Disc1 knockdown in the ganglionic eminences results in a reduced number of tangentially migrating cortical interneurons reaching the cortex [48]. In the transgenic dominant-negative Disc1 mouse model [16] and in the truncated Disc1 mouse model [49], a reduction in PV immunoreactivity in the medial prefrontal cortex (MPFC) was observed, whereas in our exploratory analysis of selected cortical ROIs we did not observe changes in the proportions of PV cells in this area in 100P mutant mice. Taken together, these results indicate that the effects of *Disc1* mutations depend critically on the exact nature of the mutation.

The first part of our study was designed to explore possible region-specific effects on subtypes of interneurons and was adequately powered to detect major differences in the PV+ interneuron population in mice carrying 100P mutations in all sensory cortical areas studied. In most cases the detectable differences were between WTs and homozygous mutants, although the results suggest that a study with a greater statistical power might show that 100P/+ has an intermediate phenotype. In the second part of our study our study was adequately powered to test answer our main hypothesis, namely that the overexpression of WT Disc1 by cortical projection neurons would increase PV+ cell density whereas expression of a 100P mutant form of Disc1 would not. We also had enough power to identify unexpected contralateral effects of WT Disc1 overexpression and to suggest effects might be layer-dependent, but these aspects of our work would benefit for further study with greater power. For example, there appeared to be a trend towards increased PV+ cell density contralaterally in fSSp and vAud but, unlike in SSp and Vis, these trends were not significant.

The fact that we did not show a loss of PV expression in MPFC contrasts with recent results showing a decrease in PV expression in this region in the 100P strain, although no change was reported in the dorsolateral frontal cortex [50]. The reasons for this might include differences in the exact regions quantified (the definition of prefrontal regions often varies between studies, [51]) or in the ages of the brains. Lee et al., (2013) [50] studied their mice at P40 whereas our study was at P21, and it is possible that the effects of 100P mutations on PV-expressing populations vary with age. This seems all the more likely since our electroporation results indicate that changes in Disc1 function can affect PV expression indirectly, presumably by influencing intercellular signalling processes that themselves vary with age for numerous reasons, such as changes in externally-induced neuronal activity. We selected P21 for our study since it is shortly before the onset of the critical period of plasticity in rodent brain and the development of PV-expressing interneurons is thought to be crucial for it to occur normally [52]. It is likely, therefore, that an abnormality of PV-expressing neurons at P21 would have a significant impact on the subsequent plasticity and functional development of the cortex with the potential to cause behavioural abnormalities such as those described by Clapcote et al. [17]

In human post-mortem studies, Reynolds and Beasley (2001) [31] showed reduced immunoreactivity to PV antibody in the prefrontal cortex of schizophrenia subjects when compared to controls. In other studies using either quantitative PCR or *in situ* hybridisation, mean PV mRNA levels were shown to be decreased not just in the prefrontal cortex but also in other cortical areas, such as anterior cingulate cortex, primary motor cortex and primary visual cortex in subjects with schizophrenia [53–54, 34]. When it comes to schizoaffective disorders in humans, prefrontal cortex is the cortical region most studied since it is believed to be intimately involved in higher cognition [55]. Since these disorders also affect sensory processing, however, we extended our analysis to cover several major areas of the sensory cortex. Our finding of widespread defects of PV expression across these regions suggests that further studies of PV neurons in these areas in humans might be fruitful.

PV interneurons are fast spiking and highly interconnected, forming large numbers of chemical and electrical synapses with other neurons. They have multiple long dendrites that can cross layers, allowing them to receive input from a large numbers of other neurons [56–58]. They receive both excitatory and inhibitory input, the latter primarily from other PV interneurons, and their axons show extensive arborisation, generating a massively divergent inhibitory output [59–61]. They are responsible for the precise control of stimulus propagation and have been implicated in the regulation of the amplitude of sensory responses, in the generation of network oscillations and in synaptic plasticity and learning [62–63]. Since proper functioning of these cells relies heavily on expression of parvalbumin, a calcium-binding protein with unique slow calcium-buffering properties [64–65], there are many reasons to anticipate that downregulation of PV across the cerebral cortex would cause a disruption of fast inhibition, loss of fine tuning in the neuronal circuitry and hence changes in behaviours such as learning and sensory responses.

The fact that PV interneurons receive extensive inputs from excitatory neurons suggests a way in which their development might be affected indirectly by changes of Disc1 function in excitatory neurons, even over long distances across the corpus callosum. It is known that the onset of PV expression in the rodent cortex during the second postnatal week is heavily dependent on neural activity [46–47]. Although Disc1 is present in both glutamatergic and GABAergic cortical neurons in an adult mouse cortex, its expression is the strongest in the cortical layers II/III and V/VI, which contain excitatory projection neurons [13]. Previous studies have suggested that Disc1 can play an important role in the establishment of excitatory neurons by regulating neurite extension [66–67], neuronal integration [68] the development of synaptic outputs of excitatory neurons [69] and dendritic complexity [70]. Moreover, as discussed above, Disc1 is likely to modulate the actions of molecules that bind to it, such as members of the Pde4 family of phosphodiesterases that can affect cAMP levels, ion channel function, nerve excitability and synaptic transmission [71–72]. It seems likely, therefore, that changes in Disc1 function in the cortical excitatory neurons would affect the activity of circuits that include afferent connections onto PV interneurons. It is possible that the indirect effects of changes in Disc1 function on PV expression observed in our study are due to alteration of cortical neural activity. If so, it might be possible, once we understand more about the nature of the activity changes, to rescue the effects of altered Disc1 function on PV interneurons by modulating activity levels in the cortex.

There are other plausible explanations for why the development of PV interneurons is affected indirectly by changes of Disc1 function in excitatory neurons. PV expression is known to be regulated cell non-autonomously by the transcription factor Otx2 during early stages of the critical period for synaptic plasticity in mouse visual cortex [73]. This study demonstrated that, in response to visual stimulation, Otx2 is transported along axons in the visual system to PV neurons via cell-to-cell transfer. Thus, the mechanism by which Disc1 function in excitatory neurons indirectly affects PV interneurons might involve an alteration in the transfer of molecules whose primary actions are on gene expression rather than on electrical activity.

In summary, we report that a specific point mutation in the N-terminus of Disc1 results in a widespread downregulation of PV in a subset of cortical interneurons across the sensory cortex. We find that altering Disc1 function selectively in cortical excitatory neurons indirectly affects cortical PV expression. We suggest that the effects of Disc1 function on PV interneurons are mediated by changes in neural activity, the intercellular transfer of signaling molecules or both. We suggest that abnormal PV neuron development in mice and humans with defective Disc1/DISC1 function is likely to contribute to behavioral anomalies.

## Supporting Information

S1 Minimal Data Set (XLSX)

S1 Table Details on ordinary one-way ANOVA with Bonferroni correction for comparison of the density of interneuronal markers in the cerebral cortex of Disc1 ENU mutants (see Fig 2) (DOCX)

S2 Table Details on multiple comparisons ANOVA with Bonferroni correction for comparison of the density of interneuronal markers across the cerebral cortex of Disc1 ENU mutants (see Fig 3). (DOCX)

S3 Table Details on multiple comparisons ANOVA with Bonferroni correction for comparison of distribution of interneuronal markers across the cortical regions of 100P cerebral cortex (see Fig 4). (DOCX)

S4 Table Details on multiple comparisons ANOVA with Bonferroni correction for comparison of the relative distributions of the electroporated cells across the cerebral cortex after an *in utero* Disc1 constructs overexpression at E14.5 (see Fig 6). (DOCX)

S5 Table Details on multiple comparisons ANOVA with Bonferroni correction for comparison of the density and distributions of the PV-positive cells across the cerebral cortex after an *in utero* Disc1 constructs overexpression at E14.5 (see Fig 7). (DOCX)
