# Supplementary material for: Altered Disrupted-in-Schizophrenia-1 Function Affects the Development of Cortical Parvalbumin Interneurons by an Indirect Mechanism
Source: PLoS One. 2016 May 31;11(5):e0156082. doi: 10.1371/journal.pone.0156082 (PMC4886955; doi:10.1371/journal.pone.0156082)
Supplement: S1 Table — (DOCX) [file pone.0156082.s002.docx]

**S1 Table.** Details on ordinary one-way ANOVA with Bonferroni correction for comparison of the density of interneuronal markers in the cerebral cortex of Disc1 ENU mutants (see Fig 2). SS – sum of squares; DF – degrees of freedom; MS – mean square; n – numerator; d – denominator.

| **ANOVA table PV expression 100P** | **SS** | **DF** | **MS** | **F (DFn, DFd)** | **P value** |
| --- | --- | --- | --- | --- | --- |
| **Treatment (between genotypes)** | 2.262 | 2 | 1.131 | F (2, 13) = 5.282 | P = 0.0209 |
| **Residual (between genotypes** | 2.784 | 13 | 0.2141 |  |  |
| **Total** | 5.046 | 15 |  |  |  |
|  |  |  |  |  |  |
| **ANOVA table GAD67 expression 100P** | **SS** | **DF** | **MS** | **F (DFn, DFd)** | **P value** |
| **Treatment (between genotypes)** | 0.03455 | 2 | 0.01728 | F (2, 9) = 0.07417 | P = 0.9291 |
| **Residual (between genotypes** | 2.096 | 9 | 0.2329 |  |  |
| **Total** | 2.131 | 11 |  |  |  |
|  |  |  |  |  |  |

| **ANOVA table STT expression 100P** | **SS** | **DF** | **MS** | **F (DFn, DFd)** | **P value** |
| --- | --- | --- | --- | --- | --- |
| **Treatment (between genotypes)** | 0.6855 | 2 | 0.3428 | F (2, 8) = 3.189 | P = 0.0959 |
| **Residual (between genotypes** | 0.8599 | 8 | 0.1075 |  |  |
| **Total** | 1.545 | 10 |  |  |  |
|  |  |  |  |  |  |
| **ANOVA table CLR expression 100P** | **SS** | **DF** | **MS** | **F (DFn, DFd)** | **P value** |
| **Treatment (between genotypes)** | 0.2072 | 2 | 0.1036 | F (2, 8) = 2.881 | P = 0.1142 |
| **Residual (between genotypes** | 0.2876 | 8 | 0.03595 |  |  |
| **Total** | 0.4948 | 10 |  |  |  |
|  |  |  |  |  |  |
| **ANOVA table PV expression 31L** | **SS** | **DF** | **MS** | **F (DFn, DFd)** | **P value** |
| **Treatment (between genotypes)** | 0.02995 | 2 | 0.01498 | F (2, 16) = 0.07371 | P = 0.9293 |
| **Residual (between genotypes** | 3.251 | 16 | 0.2032 |  |  |
| **Total** | 3.281 | 18 |  |  |  |
|  |  |  |  |  |  |
| **ANOVA table GAD67 expression 31L** | **SS** | **DF** | **MS** | **F (DFn, DFd)** | **P value** |
| **Treatment (between genotypes)** | 1.576 | 2 | 0.7879 | F (2, 8) = 1.762 | P = 0.2323 |
| **Residual (between genotypes** | 3.578 | 8 | 0.4473 |  |  |
| **Total** | 5.154 | 10 |  |  |  |
|  |  |  |  |  |  |
| **ANOVA table STT expression 31L** | **SS** | **DF** | **MS** | **F (DFn, DFd)** | **P value** |
| **Treatment (between genotypes)** | 0.2517 | 2 | 0.1259 | F (2, 15) = 1.691 | P = 0.2176 |
| **Residual (between genotypes** | 1.116 | 15 | 0.07443 |  |  |
| **Total** | 1.368 | 17 |  |  |  |
|  |  |  |  |  |  |
| **ANOVA table CLR expression 31L** | **SS** | **DF** | **MS** | **F (DFn, DFd)** | **P value** |
| **Treatment (between genotypes)** | 0.1987 | 2 | 0.09937 | F (2, 8) = 3.053 | P = 0.1035 |
| **Residual (between genotypes** | 0.2604 | 8 | 0.03255 |  |  |
| **Total** | 0.4591 | 10 |  |  |  |
