# Supplementary material for: Altered Disrupted-in-Schizophrenia-1 Function Affects the Development of Cortical Parvalbumin Interneurons by an Indirect Mechanism
Source: PLoS One. 2016 May 31;11(5):e0156082. doi: 10.1371/journal.pone.0156082 (PMC4886955; doi:10.1371/journal.pone.0156082)
Supplement: S2 Table — (DOCX) [file pone.0156082.s003.docx]

**S2 Table.** Details on two-way ANOVA with Dunnet’s correction for comparison of the density of interneuronal markers across the cerebral cortex of Disc1 ENU mutants (see Fig 3). SS – sum of squares; DF – degrees of freedom; MS – mean square; n – numerator; d – denominator.

| **ANOVA table PV expression 100P** | **SS** | **DF** | | **MS** | **F (DFn, DFd)** | **P value** |
| --- | --- | --- | --- | --- | --- | --- |
| **Interaction** | 3.333 | 8 | | 0.4166 | F (8, 56) = 1.607 | P = 0.1440 |
| **Cortical region Factor** | 34.46 | 4 | | 8.614 | F (4, 56) = 33.32 | P < 0.0001 |
| **Genotype Factor** | 6.128 | 2 | | 3.064 | F (2, 56) = 11.82 | P < 0.0001 |
| **Residual** | 14.26 | 55 | | 0.2592 |  |  |
|  |  |  | |  |  |  |
| **ANOVA table PV mRNA** | **SS** | **DF** | | **MS** | **F (DFn, DFd)** | **P value** |
| **Interaction** | 0.2350 | 6 | | 0.03916 | F (6, 36) = 0.07409 | P = 0.9982 |
| **Cortical region Factor** | 35.94 | 3 | | 11.98 | F (3, 36) = 22.67 | P < 0.0001 |
| **Genotype Factor** | 14.17 | 2 | | 7.083 | F (2, 36) = 13.40 | P < 0.0001 |
| **Residual** | 19.03 | 36 | | 0.5285 |  |  |
|  |  |  | |  |  |  |
| **ANOVA table GAD67 expression 100P** | **SS** | **DF** | | **MS** | **F (DFn, DFd)** | **P value** |
| **Interaction** | 7.861 | 6 | | 1.310 | F (6, 34) = 1.078 | P = 0.3950 |
| **Cortical region Factor** | 12.73 | 3 | | 4.244 | F (3, 34) = 3.491 | P = 0.0260 |
| **Genotype Factor** | 1.444 | 2 | | 0.7219 | F (2, 34) = 0.5938 | P = 0.5578 |
| **Residual** | 41.33 | 34 | | 1.216 |  |  |
|  |  |  | |  |  |  |
| **ANOVA table STT expression 100P** | **SS** | **DF** | | **MS** | **F (DFn, DFd)** | **P value** |
| **Interaction** | 2.712 | 6 | | 0.4520 | F (6, 29) = 2.149 | P = 0.0777 |
| **Cortical region Factor** | 3.082 | 3 | | 1.027 | F (3, 29) = 4.885 | P = 0.0072 |
| **Genotype Factor** | 0.5333 | 2 | | 0.2666 | F (2, 29) = 1.268 | P = 0.2965 |
| **Residual** | 6.098 | 29 | | 0.2103 |  |  |
|  |  |  | |  |  |  |
| **ANOVA table CLR expression 100P** | **SS** | | **DF** | **MS** | **F (DFn, DFd)** | **P value** |
| **Interaction** | 0.9080 | | 6 | 0.1513 | F (6, 30) = 1.291 | P = 0.2912 |
| **Cortical region Factor** | 11.64 | | 3 | 3.878 | F (3, 30) = 33.09 | P < 0.0001 |
| **Genotype Factor** | 0.2181 | | 2 | 0.1091 | F (2, 30) = 0.9305 | P = 0.4055 |
| **Residual** | 3.516 | | 30 | 0.1172 |  |  |
|  |  | |  |  |  |  |
| **ANOVA table PV expression 31L** | **SS** | **DF** | | **MS** | **F (DFn, DFd)** | **P value** |
| **Interaction** | 1.359 | 6 | | 0.2266 | F (6, 57) = 0.7267 | P = 0.6299 |
| **Cortical region Factor** | 31.16 | 3 | | 10.39 | F (3, 57) = 33.31 | P < 0.0001 |
| **Genotype Factor** | 0.3115 | 2 | | 0.1557 | F (2, 57) = 0.4995 | P = 0.6094 |
| **Residual** | 17.77 | 57 | | 0.3118 |  |  |
|  |  |  | |  |  |  |
| **ANOVA table GAD67 expression 31L** | **SS** | **DF** | | **MS** | **F (DFn, DFd)** | **P value** |
| **Interaction** | 4.988 | 6 | | 0.8314 | F (6, 33) = 0.6212 | P = 0.7119 |
| **Cortical region Factor** | 12.45 | 3 | | 4.150 | F (3, 33) = 3.101 | P = 0.0399 |
| **Genotype Factor** | 8.130 | 2 | | 4.065 | F (2, 33) = 3.037 | P = 0.0616 |
| **Residual** | 44.17 | 33 | | 1.338 |  |  |
|  |  |  | |  |  |  |
| **ANOVA table CLR expression 31L** | **SS** | **DF** | | **MS** | **F (DFn, DFd)** | **P value** |
| **Interaction** | 0.5234 | 6 | | 0.08724 | F (6, 34) = 0.8276 | P = 0.5568 |
| **Cortical region Factor** | 18.69 | 3 | | 6.230 | F (3, 34) = 59.10 | P < 0.0001 |
| **Genotype Factor** | 0.4158 | 2 | | 0.2079 | F (2, 34) = 1.972 | P = 0.1547 |
| **Residual** | 3.584 | 34 | | 0.1054 |  |  |
|  |  |  | |  |  |  |
| **ANOVA table STT expression 31L** | **SS** | **DF** | | **MS** | **F (DFn, DFd)** | **P value** |
| **Interaction** | 1.542 | 6 | | 0.2569 | F (6, 56) = 1.803 | P = 0.1151 |
| **Cortical region Factor** | 1.274 | 3 | | 0.4248 | F (3, 56) = 2.981 | P = 0.0390 |
| **Genotype Factor** | 0.1526 | 2 | | 0.07630 | F (2, 56) = 0.5355 | P = 0.5883 |
| **Residual** | 7.979 | 56 | | 0.1425 |  |  |
