# Supplementary material for: Altered Disrupted-in-Schizophrenia-1 Function Affects the Development of Cortical Parvalbumin Interneurons by an Indirect Mechanism
Source: PLoS One. 2016 May 31;11(5):e0156082. doi: 10.1371/journal.pone.0156082 (PMC4886955; doi:10.1371/journal.pone.0156082)
Supplement: S3 Table — (DOCX) [file pone.0156082.s004.docx]

**S3 Table.** Details on two-way ANOVA with Dunnet’s correction for comparison of distribution of interneuronal markers across the cortical regions of 100P cerebral cortex (see Fig 4). SS – sum of squares; DF – degrees of freedom; MS – mean square; n – numerator; d - denominator

| **ANOVA table PV-cells distribution fSSp** | **SS** | **DF** | **MS** | **F (DFn, DFd)** | **P value** |
| --- | --- | --- | --- | --- | --- |
| **Interaction** | 12.57 | 8 | 1.571 | F (8, 65) = 1.396 | P = 0.2152 |
| **Cortical layer Factor** | 271.9 | 4 | 67.97 | F (4, 65) = 60.39 | P < 0.0001 |
| **Genotype Factor** | 13.06 | 2 | 6.529 | F (2, 65) = 5.801 | P = 0.0048 |
| **Residual** | 73.16 | 65 | 1.126 |  |  |
|  |  |  |  |  |  |
| **ANOVA table PV-cells distribution SSp** | **SS** | **DF** | **MS** | **F (DFn, DFd)** | **P value** |
| **Interaction** | 16.80 | 8 | 2.100 | F (8, 65) = 3.225 | P = 0.0038 |
| **Cortical layer Factor** | 154.5 | 4 | 38.63 | F (4, 65) = 59.34 | P < 0.0001 |
| **Genotype Factor** | 15.92 | 2 | 7.959 | F (2, 65) = 12.22 | P < 0.0001 |
| **Residual** | 42.32 | 65 | 0.6510 |  |  |
|  |  |  |  |  |  |
| **ANOVA table PV-cells distribution Aud** | **SS** | **DF** | **MS** | **F (DFn, DFd)** | **P value** |
| **Interaction** | 22.49 | 8 | 2.811 | F (8, 65) = 4.954 | P < 0.0001 |
| **Cortical layer Factor** | 89.57 | 4 | 22.39 | F (4, 65) = 39.46 | P < 0.0001 |
| **Genotype Factor** | 19.05 | 2 | 9.527 | F (2, 65) = 16.79 | P < 0.0001 |
| **Residual** | 36.88 | 65 | 0.5674 |  |  |
|  |  |  |  |  |  |
| **ANOVA table PV-cells distribution Vis** | **SS** | **DF** | **MS** | **F (DFn, DFd)** | **P value** |
| **Interaction** | 13.31 | 8 | 1.664 | F (8, 35) = 3.721 | P = 0.0031 |
| **Cortical layer Factor** | 157.3 | 4 | 39.32 | F (4, 35) = 87.93 | P < 0.0001 |
| **Genotype Factor** | 6.417 | 2 | 3.208 | F (2, 35) = 7.174 | P = 0.0024 |
| **Residual** | 15.65 | 35 | 0.4472 |  |  |
|  |  |  |  |  |  |
| **ANOVA table interneurons distribution fSSp** | **SS** | **DF** | **MS** | **F (DFn, DFd)** | **P value** |
| **Interaction** | 31.53 | 8 | 3.941 | F (8, 45) = 0.5412 | P = 0.8191 |
| **Cortical layer Factor** | 1471 | 4 | 367.7 | F (4, 45) = 50.50 | P < 0.0001 |
| **Genotype Factor** | 5.666 | 2 | 2.833 | F (2, 45) = 0.3890 | P = 0.6800 |
| **Residual** | 327.7 | 45 | 7.282 |  |  |
|  |  |  |  |  |  |
| **ANOVA table interneurons distribution SSp** | **SS** | **DF** | **MS** | **F (DFn, DFd)** | **P value** |
| **Interaction** | 90.05 | 8 | 11.26 | F (8, 45) = 2.051 | P = 0.0615 |
| **Cortical layer Factor** | 885.3 | 4 | 221.3 | F (4, 45) = 40.32 | P < 0.0001 |
| **Genotype Factor** | 10.85 | 2 | 5.427 | F (2, 45) = 0.9888 | P = 0.3800 |
| **Residual** | 247.0 | 45 | 5.489 |  |  |
|  |  |  |  |  |  |
| **ANOVA table interneurons distribution Aud** | **SS** | **DF** | **MS** | **F (DFn, DFd)** | **P value** |
| **Interaction** | 782.2 | 8 | 97.78 | F (8, 45) = 6.551 | P < 0.0001 |
| **Cortical layer Factor** | 1816 | 4 | 454.0 | F (4, 45) = 30.41 | P < 0.0001 |
| **Genotype Factor** | 99.79 | 2 | 49.90 | F (2, 45) = 3.343 | P = 0.0443 |
| **Residual** | 671.7 | 45 | 14.93 |  |  |
|  |  |  |  |  |  |
| **ANOVA table interneurons distribution Vis** | **SS** | **DF** | **MS** | **F (DFn, DFd)** | **P value** |
| **Interaction** | 30.73 | 8 | 3.841 | F (8, 35) = 0.3776 | P = 0.9253 |
| **Cortical layer Factor** | 1589 | 4 | 397.2 | F (4, 35) = 39.04 | P < 0.0001 |
| **Genotype Factor** | 2.752 | 2 | 1.376 | F (2, 35) = 0.1353 | P = 0.8739 |
| **Residual** | 356.0 | 35 | 10.17 |  |  |
