# Supplementary material for: Altered Disrupted-in-Schizophrenia-1 Function Affects the Development of Cortical Parvalbumin Interneurons by an Indirect Mechanism
Source: PLoS One. 2016 May 31;11(5):e0156082. doi: 10.1371/journal.pone.0156082 (PMC4886955; doi:10.1371/journal.pone.0156082)
Supplement: S4 Table — (DOCX) [file pone.0156082.s005.docx]

**S4 Table.** Details on two-way ANOVA with Bonferroni correction for comparison of the relative distributions of the electroporated cells across the cerebral cortex after an *in utero* Disc1 constructs overexpression at E14.5 (see Fig 6). SS – sum of squares; DF – degrees of freedom; MS – mean square; n – numerator; d - denominator

| **ANOVA table GFP distribution fSSp cortex** | **SS** | **DF** | **MS** | **F (DFn, DFd)** | **P value** |
| --- | --- | --- | --- | --- | --- |
| **Interaction** | 725.7 | 8 | 90.71 | F (8, 90) = 0.9398 | P = 0.4881 |
| **Cortical layer Factor** | 57229 | 4 | 14307 | F (4, 90) = 148.2 | P < 0.0001 |
| **Plasmid Factor** | 0.0 | 2 | 0.0 | F (2, 90) = 0.0 | P > 0.9999 |
| **Residual** | 8686 | 90 | 96.51 |  |  |
|  |  |  |  |  |  |
| **ANOVA table GFP distribution SSp cortex** | **SS** | **DF** | **MS** | **F (DFn, DFd)** | **P value** |
| **Interaction** | 3010 | 8 | 376.3 | F (8, 90) = 3.580 | P = 0.0012 |
| **Cortical layer Factor** | 52165 | 4 | 13041 | F (4, 90) = 124.1 | P < 0.0001 |
| **Plasmid Factor** | 0.0 | 2 | 0.0 | F (2, 90) = 0.0 | P > 0.9999 |
| **Residual** | 9459 | 90 | 105.1 |  |  |
|  |  |  |  |  |  |
| **ANOVA table GFP distribution Aud cortex** | **SS** | **DF** | **MS** | **F (DFn, DFd)** | **P value** |
| **Interaction** | 574.0 | 8 | 71.74 | F (8, 45) = 0.5404 | P = 0.8197 |
| **Cortical layer Factor** | 67909 | 4 | 16977 | F (4, 45) = 127.9 | P < 0.0001 |
| **Plasmid Factor** | 0.8681 | 2 | 0.4340 | F (2, 45) = 0.003269 | P = 0.9967 |
| **Residual** | 5974 | 45 | 132.8 |  |  |
|  |  |  |  |  |  |
| **ANOVA table GFP distribution Vis cortex** | **SS** | **DF** | **MS** | **F (DFn, DFd)** | **P value** |
| **Interaction** | 1025 | 8 | 128.1 | F (8, 45) = 3.037 | P = 0.0081 |
| **Cortical layer Factor** | 29466 | 4 | 7366 | F (4, 45) = 174.7 | P < 0.0001 |
| **Plasmid Factor** | 0.0 | 2 | 0.0 | F (2, 45) = 0.0 | P >0.9999 |
| **Residual** | 1898 | 45 | 42.17 |  |  |
