# Supplementary material for: Altered Disrupted-in-Schizophrenia-1 Function Affects the Development of Cortical Parvalbumin Interneurons by an Indirect Mechanism
Source: PLoS One. 2016 May 31;11(5):e0156082. doi: 10.1371/journal.pone.0156082 (PMC4886955; doi:10.1371/journal.pone.0156082)
Supplement: S5 Table — (DOCX) [file pone.0156082.s006.docx]

**S5 Table.** Details on two-way ANOVA with Bonferroni correction for comparison of the density and distributions of the PV-positive cells across the cerebral cortex after an *in utero* Disc1 constructs overexpression at E14.5 (see Fig 7). SS – sum of squares; DF – degrees of freedom; MS – mean square; n – numerator; d – denominator.

| **ANOVA table PV-cells density, ipsilateral side** | **SS** | **DF** | **MS** | **F (DFn, DFd)** | **P value** |
| --- | --- | --- | --- | --- | --- |
| **Interaction** | 2314 | 8 | 289.2 | F (8, 60) = 1.033 | P = 0.4220 |
| **Cortical region Factor** | 11519 | 4 | 2880 | F (4, 60) = 10.28 | P < 0.0001 |
| **Plasmid Factor** | 11385 | 2 | 5693 | F (2, 60) = 20.33 | P < 0.0001 |
| **Residual** | 16803 | 60 | 280.0 |  |  |
|  |  |  |  |  |  |
| **ANOVA table PV-cells density, contralateral side** | **SS** | **DF** | **MS** | **F (DFn, DFd)** | **P value** |
| **Interaction** | 1442 | 6 | 240.3 | F (6, 54) = 0.7985 | P = 0.5753 |
| **Cortical region Factor** | 18072 | 3 | 6024 | F (3, 54) = 20.02 | P < 0.0001 |
| **Plasmid Factor** | 5457 | 2 | 2728 | F (2, 54) = 9.066 | P = 0.0004 |
| **Residual** | 16251 | 54 | 300.9 |  |  |
|  |  |  |  |  |  |
| **ANOVA table PV-cells distribution SSp ipsi.** | **SS** | **DF** | **MS** | **F (DFn, DFd)** | **P value** |
| **Interaction** | 19589 | 8 | 2449 | F (8, 90) = 2.627 | P = 0.0124 |
| **Cortical layer Factor** | 422893 | 4 | 105723 | F (4, 90) = 113.4 | P < 0.0001 |
| **Plasmid Factor** | 35562 | 2 | 17781 | F (2, 90) = 19.08 | P < 0.0001 |
| **Residual** | 83889 | 90 | 932.1 |  |  |
|  |  |  |  |  |  |
| **ANOVA table PV-cells distribution SSp contra.** | **SS** | **DF** | **MS** | **F (DFn, DFd)** | **P value** |
| **Interaction** | 7300 | 8 | 912.5 | F (8, 90) = 1.174 | P = 0.3233 |
| **Cortical layer Factor** | 477951 | 4 | 119488 | F (4, 90) = 153.8 | P < 0.0001 |
| **Plasmid Factor** | 21627 | 2 | 10814 | F (2, 90) = 13.92 | P < 0.0001 |
| **Residual** | 69934 | 90 | 777.0 |  |  |
|  |  |  |  |  |  |
| **ANOVA table PV-cells distribution Vis ipsi** | **SS** | **DF** | **MS** | **F (DFn, DFd)** | **P value** |
| **Interaction** | 14920 | 8 | 1865 | F (8, 45) = 3.134 | P = 0.0067 |
| **Cortical layer Factor** | 237103 | 4 | 59276 | F (4, 45) = 99.62 | P < 0.0001 |
| **Plasmid Factor** | 12560 | 2 | 6280 | F (2, 45) = 10.55 | P = 0.0002 |
| **Residual** | 26777 | 45 | 595.0 |  |  |
|  |  |  |  |  |  |
| **ANOVA table PV-cells distribution Vis contra** | **SS** | **DF** | **MS** | **F (DFn, DFd)** | **P value** |
| **Interaction** | 6925 | 8 | 865.6 | F (8, 45) = 1.042 | P = 0.4199 |
| **Cortical layer Factor** | 207846 | 4 | 51961 | F (4, 45) = 62.54 | P < 0.0001 |
| **Plasmid Factor** | 7806 | 2 | 3903 | F (2, 45) = 4.697 | P = 0.0140 |
| **Residual** | 37388 | 45 | 830.8 |  |  |
